# Polydatin Protects Diabetic Heart against Ischemia-Reperfusion Injury via Notch1/Hes1-Mediated Activation of Pten/Akt Signaling

**DOI:** 10.1155/2018/2750695

**Published:** 2018-02-13

**Authors:** Liming Yu, Zhi Li, Xue Dong, Xiaodong Xue, Yu Liu, Shu Xu, Jian Zhang, Jinsong Han, Yang Yang, Huishan Wang

**Affiliations:** ^1^Department of Cardiovascular Surgery, General Hospital of Shenyang Military Region, 83 Wenhua Road, Shenyang, Liaoning 110016, China; ^2^Department of Pharmacy, General Hospital of Shenyang Military Region, 83 Wenhua Road, Shenyang, Liaoning 110016, China; ^3^Department of Neurosurgery, General Hospital of Shenyang Military Region, 83 Wenhua Road, Shenyang, Liaoning 110016, China; ^4^Faculty of Life Science, Northwest University, 229 Taibai North Road, Xi'an, Shaanxi 710069, China; ^5^Department of Biomedical Engineering, The Fourth Military Medical University, 169 Changle West Road, Xi'an, Shaanxi 710032, China

## Abstract

Diabetes exacerbates oxidative/nitrative stress during myocardial ischemia-reperfusion (MI/R) injury. Recent studies highlighted the cardioprotective actions of polydatin. However, its effect on diabetic MI/R injury and the underlying mechanisms remain unknown. This work was undertaken to evaluate the effect of polydatin on diabetic MI/R injury with a focus on Notch1/Hes1 signaling and myocardial oxidative/nitrative stress. Streptozotocin- (STZ-) induced diabetic rats were administered with polydatin (20 mg/kg/d) in the absence or presence of DAPT (a *γ*-secretase inhibitor) or LY294002 (a PI3K/Akt inhibitor) and then subjected to MI/R injury. Polydatin administration preserved cardiac function and reduced myocardial infarct size. Moreover, polydatin ameliorated myocardial oxidative/nitrative stress damage as evidenced by decreased myocardial superoxide generation, malondialdehyde, gp91*^phox^* expression, iNOS expression, NO metabolite level, and nitrotyrosine content and increased eNOS phosphorylation. However, these effects were blocked by DAPT administration. DAPT also inhibited the stimulatory effect of polydatin on the Notch1/Hes1-Pten/Akt signaling pathway in a diabetic myocardium. Additionally, LY294002 not only abolished polydatin's antiapoptotic effect but also reversed its inhibitory effect on myocardial oxidative/nitrative stress. Polydatin effectively reduced MI/R injury and improved left ventricular functional recovery under diabetic condition by ameliorating oxidative/nitrative stress damage. Importantly, Notch1/Hes1-mediated activation of Pten/Akt signaling played a crucial role in this process.

## 1. Introduction

Patients with diabetes mellitus (DM) are more susceptible to ischemic heart disease and sustain a more unfavorable prognosis than nondiabetic individuals, despite the great advances in pharmacological therapies and surgical techniques in recent years [1–3]. To make things worse, it has been shown that diabetes mellitus even compromises the effectiveness of various cardioprotective interventions [4]. Previously, we and others have found that hyperglycemia results in myocardial oxidative stress and nitrative stress damage by stimulating reactive oxygen species (ROS) production and breaking the balance between nitric oxide (NO) and O_2_^−^ [5–7]. Furthermore, the oxidative/nitrative stress is markedly aggravated in the presence of diabetes following myocardial ischemia-reperfusion (MI/R) injury and eventually exacerbates MI/R injury [8]. Developing adjunctive strategy to suppress oxidative/nitrative stress damage and increasing the beneficial effect of reperfusion therapy are of great clinical importance.

The Notch pathway has been recognized as an evolutionarily conserved signaling which regulates cell homeostasis and tissue formation during embryonic and adult life [9]. It is a family of transmembrane receptors that, once bound to one of its ligands, undergo proteolytic cleavages by TNF-*α*-converting enzyme (TACE) and the *γ*-secretase complex. The released intracellular domain of Notch (Notch ICD) then translocates into the nucleus and regulates the target genes Hairy and enhancer of split (Hes) and Hey transcription [9, 10]. Our previous studies showed that pharmacological activation of Notch1/Hes1 signaling inhibited myocardial oxidative stress damage and preserved heart function during MI/R injury [11, 12]. Additionally, Pei et al. found that knockdown of Notch1 exacerbated cardiac damage following MI/R injury by enhancing oxidative and nitrative stress [13]. However, under diabetic condition, whether Notch1 signaling exerts a similar cardioprotective effect and the underlying mechanisms remains unknown.

Polydatin (3,4′,5-trihydroxystilbene-3-*β*-D-glucoside, PD) is a natural hydroxyl-diphenyl ethylene compound isolated from the perennial herbage *Polygonum cuspidatum* Sieb. et Zucc [14, 15]. Modern pharmacological studies have shown that polydatin regulates multiple biological activities of cardiovascular systems, including antioxidation, anti-inflammation, promotion of microcirculation, and reduction of lipid synthesis [15, 16]. Specifically, during the period of ischemia-reperfusion, polydatin exerted a potential protective effect by either promoting autophagic flux and myocardial Ca^2+^ handling [17, 18] or regulating classic intracellular signaling including renin-angiotensin system (RAS) and the downstream Rho kinase (ROCK) pathway [19]. Interestingly, one study by Huang et al. demonstrated that polydatin attenuated diabetic myocardial hypertrophy and inhibited nuclear factor-kappa B (NF-*κ*B), cyclooxygenase-2 (COX-2), and inducible NO synthase (iNOS) signaling pathways [20], indicating that polydatin might also exert cardioprotection in diabetic setting. However, its effect on diabetic MI/R injury and the regulatory role of polydatin on oxidative/nitrative stress are still poorly understood. Additionally, the effect of polydatin on Notch1/Hes1 signaling is also not clear.

On the basis of the above observations, we employed streptozotocin-induced diabetic animal model and coronary artery ligation-induced MI/R model to (1) investigate whether polydatin reduces MI/R injury in diabetic setting, (2) determine the regulatory role of polydatin on myocardial oxidative stress and nitrative stress damage, and (3) explore the detailed role of Notch1/Hes1 signaling during this process.

## 2. Materials and Methods

### 2.1. Animals

Adult male Sprague-Dawley (SD) rats (weighing 220-250 g) were supplied by the Experimental Animal Center of the General Hospital of Shenyang Military Region, Shenyang, China. The rats were housed 3 rats per cage in a quiet laboratory room kept at constant temperature and humidity under a 12 h dark/light cycle. The animals were allowed free access to standard rodent chow and distilled water. All surgical procedures were performed according to the Guide for the Care and Use of Laboratory Animals published by the US National Institutes of Health (NIH Publication number 85-23, revised 1996). This experiment was approved by the Animal Care Committee of the General Hospital of Shenyang Military Region.

### 2.2. Induction of Diabetes

As described in our previous study [21, 22], streptozotocin (STZ, Sigma-Aldrich, MO, USA, 50 mg/kg) was injected to the rats through an abdominal cavity for 3 consecutive days to induce a diabetic model. After 7 days, the tail blood was collected and the animals with fasting plasma glucose (FBG) ≥ 11.1 mmol/L were selected as the diabetes model. Intraperitoneal glucose tolerance test (IPGTT) and oral glucose tolerance test (OGTT) were performed by administering glucose (2 g/kg, gastric lavage or intraperitoneal injection) to further confirm the diabetic model as described in our previous studies [21]. Plasma glucose level was measured at 0 (before glucose load), 30, 60, 90, and 120 min.

### 2.3. Ischemia-Reperfusion Procedure

After the induction of diabetes, 210 SD rats were randomly assigned to the following groups: the sham group, which received the same operation except that the suture around the left anterior descending coronary artery was left untied; the MI/R + V group, which was orally treated with vehicle (distilled water, 0.5 mL/d) for 3 consecutive days and once again right before the MI/R operation; the MI/R + PD group, which was orally treated with polydatin (PD, Aladdin Chemicals, Shanghai, China; 20 mg/kg/d) for 3 consecutive days and once again right before the MI/R operation; the MI/R + PD + DAPT group, which was treated with polydatin (as above) and DAPT (Santa Cruz, CA, USA; 50 mg/kg, intraperitoneally, 20 min before the beginning of myocardial reperfusion); the MI/R + PD + LY group, which was treated with polydatin (as above) and LY294002 (LY, Santa Cruz, CA, USA; 30 mg/kg, intraperitoneally, 20 min before the beginning of myocardial reperfusion); the sham + DAPT group, which received sham operation and DAPT injection (as above); and the sham + LY group, which received sham operation and LY injection (as above). The dosages of polydatin and exogenous inhibitors were chosen based on previous publications [11, 12, 18, 19, 23, 24]. MI/R surgery was carried out as described previously [23]. The experimental animals were intraperitoneally anesthetized (sodium pentobarbital, 40 mg/kg) and ventilated on a rodent ventilator (Taimeng Technology, Chengdu, China) via a tracheal intubation. A left lateral thoracotomy was performed to expose the heart. The left anterior descending coronary artery was ligated with a prolene monofilament suture (7–0, Ethicon) at about 2 mm from its origin. Then, the suture was passed through a short piece of rubber tube to create a reversible snare. Myocardial ischemia was initiated by clamping the snare onto the epicardial surface tightly. After 30 min of ischemia, the snare was loosened, and the heart was reperfused for 3 h (for the analysis of cardiac function and protein expressions) or 6 h (for the analysis of apoptosis and infarct size). During the operation, the cardiac function was determined using a hemodynamic monitoring system (Taimeng Technology, Chengdu, China). Left ventricular systolic pressure (LVSP) and first derivative of left ventricular pressure (+dP/dt_max_ and −dP/dt_max_) were directly monitored and calculated by computer algorithms.

### 2.4. Determination of Myocardial Infarct Size and Apoptosis

Myocardial infarct size was determined by the use of Evans blue-TTC double staining (Solarbio Technology, Beijing, China) as described in our previous study [6]. The viable area, area at risk (AAR), and infarct area (INF) were captured digitally and analyzed using Image-Pro Plus software (Media Cybernetics, MA, USA). The result was expressed as the percentage of the infarct area over the total area at risk (INF/AAR × 100%). Myocardial apoptosis was assessed by the use of a terminal deoxynucleotidyl transferase-mediated dUTP nick-end labeling (TUNEL) kit (Roche, Mannheim, Germany), as described previously [23]. Anti-*α*-sarcomeric actin antibody (Abcam, Cambridge, UK) was used to stain cardiomyocytes. The result was expressed by the number of apoptotic myocytes/the total number of myocytes counted × 100%.

### 2.5. Determination of Plasma Creatine Kinase and Lactate Dehydrogenase

At the end of the reperfusion, blood samples (1 mL) were collected and centrifuged for plasma separation (1000*g*, 15 min, 4°C). Plasma creatine kinase (CK) and lactate dehydrogenase (LDH) activities were measured using commercial assay kits (Jiancheng Biotechnology, Nanjing, China) according to the manufacturer's instructions [5]. All measurements were carried out in duplicate.

### 2.6. Quantification of Superoxide Generation, Malondialdehyde, and Superoxide Dismutase

Superoxide generation in the myocardial tissue of LV was measured by the use of a lucigenin-enhanced chemiluminescence kit (Jiancheng Biotechnology, Nanjing, China) as described previously [22]. The results were expressed as relative light units (RLU) per second per milligram protein weight (RLU/mg/s). The malondialdehyde (MDA) level and activities of antioxidant superoxide dismutase (SOD) were determined using spectrophotometric assay kits (Jiancheng Biotechnology, Nanjing, China) as described in our previous studies [22].

### 2.7. Determination of Total Nitric Oxide and Nitrotyrosine Content

At the end of the reperfusion, the cardiac tissues of LV were rinsed and homogenized. Two major in vivo metabolites of NO, nitrite (NO2^−^), and nitrate (NO3^−^) representing overall NO production in the myocardium were measured by the use of nitrate reductase kits (Jiancheng Biotechnology, Nanjing, China) according to the manufacturer's instructions [13]. As a key oxidative/nitrative stress marker and the footprint of myocardial peroxynitrite formation, nitrotyrosine content was determined using a commercially available enzyme-linked immunosorbent assay kit (Millipore, Billerica, MA, USA) as described previously [25].

### 2.8. Immunohistochemistry Staining

The nitrotyrosine content was also measured by immunohistochemistry staining. The detailed protocol was described in our previous publications [21, 22]. The LV tissues were fixed with 4% paraformaldehyde and embedded in paraffin. Then, they were cut into 3 mm thickness and stained with primary antibody (anti-nitrotyrosine antibody, Cell Signaling Technology, MA, USA, 1 : 200 dilution). Then, the sections were incubated with horseradish peroxidase- (HRP-) conjugated secondary antibodies (Zhongshan Biotechnology, Beijing, China) and detected with 3,3′-diaminobenzidine (DAB) staining (Zhongshan Biotechnology, Beijing, China). Five fields of each section were randomly chosen and photographed at ×200 magnification (Olympus BX-63, Tokyo, Japan). The graphs were analyzed and calculated using Image-Pro Plus software (Media Cybernetics, MA, USA).

### 2.9. Nuclear Fraction Extraction

The nuclear fraction of the myocardial tissue was separated and prepared as described in our previous publication [22]. The myocardial tissue was lysed and suspended in homogenization buffer [140 NaCl, 20 Tris (pH 7.9), 1.5 MgCl_2_, 1 EGTA, 1 EDTA, 1 DTT, 0.5 PMSF (mmol/L), protease inhibitor, and 0.5% (*w*/*v*) NP-40]. The homogenates were centrifuged (5000 rpm, 10 min), and the supernatant (cytoplasmic fraction) was stored at −80°C. The pellets were resuspended in nuclear extraction buffer [60 KCl, 50 Tris (pH 7.9), 2 DTT, 1 EDTA, 1 EGTA, 1 PMSF (mmol/L), and protease inhibitor]. Then, the suspension was centrifuged (13,000 rpm, 15 min). The supernatant (nuclear fraction) was collected and stored at −80°C for further assessment.

### 2.10. Immunofluorescent Staining

Immunofluorescent staining was performed as described in our previous publication [12, 23]. Briefly, the paraffin-embedded heart sections (3 *μ*m) were incubated in 1% normal donkey serum in PBS containing 0.3% Triton X-100 (1 h, 37°C). The primary antibodies to Notch1 ICD and *α*-sarcomeric actin (Abcam, Cambridge, UK, 1 : 50 dilution) were added and incubated with the sections overnight (4°C). Then, the paraffin-embedded heart sections were washed with PBS 3 times and incubated with Cy3-conjugated goat anti-rabbit IgG and FITC-conjugated donkey anti-goat IgG (Abbkine, Redlands, CA, USA). 4′,6-Diamidino-2-phenylindole (DAPI, Sigma-Aldrich, MO, USA) was used to stain the nuclei. The cellular distribution of Notch1 ICD and *α*-sarcomeric actin was observed using a confocal microscope (FV1000, Olympus, Tokyo, Japan). Five fields of each section were randomly chosen and photographed. The graphs were analyzed and calculated using Image-Pro Plus software (Media Cybernetics, MA, USA).

### 2.11. Real-Time PCR

Total RNA was extracted from flash-frozen myocardial tissue using TRIzol reagent (Invitrogen, Carlsbad, CA, USA) according to the manufacturer's protocol. cDNA was synthesized from 2 *μ*g RNA via a reverse transcription reagent kit (TaKaRa, Japan) following the manufacturer's instruction. The resulting cDNA was used as a template for qRT-PCR assay using specific Pten primer and SYBR Green reagent (TaKaRa, Japan) in the StepOne Plus Real-time PCR System (Applied Biosystems, USA). The PCR condition was programmed as follows: initial denaturation at 95°C for 3 min, followed by 40 amplification cycles of 95°C for 5 sec, and annealing at 60°C for 30 sec. *β*-Actin served as an endogenous control. The primer sequences employed in this study were as follows: Pten—forward 5′-ATACCAGGACCAGAGGAAACC-3′ and reverse 5′-TTGTCATTATCCGCACGCTC-3′—and *β*-actin—forward 5′-CGTTGACATCCGTAAAGAC-3′ and reverse 5′-TAGGAGCCAGGGCAGTA-3′. All reactions were carried out in triplicate. The relative Pten expressions of the experimental groups were all normalized to the sham group.

### 2.12. Western Blot Analysis

Western blot was performed as described in our previous publications [21]. In brief, the myocardial tissues were lysed with ice-cold RIPA buffer (Beyotime Biotechnology, Shanghai, China) containing 1% protease inhibitor cocktail (Sigma-Aldrich, MO, USA). After protein concentration measurement by the modified Bradford assay (Bio-Rad Laboratories, Hercules, CA, USA), the proteins were separated by SDS-polyacrylamide gel electrophoresis and transferred onto nitrocellulose membranes. Then, they were probed with antibodies against cleaved caspase-3, gp91*^phox^*, iNOS, p-eNOS, eNOS, Notch1 ICD, PCNA, Hes1, Pten, p-Akt, Akt, and *β*-actin (1 : 1000, Cell Signaling Technology, MA, USA) overnight (4°C) followed by incubation with HRP-conjugated secondary antibodies (1 : 5000 Zhongshan Biotechnology, Beijing, China) for 1 h (room temperature). The SuperSignal ECL kit (Thermo Fisher Scientific, Rockville, MD, USA) was employed to detect the antigen-antibody complexes. The bands were quantified and analyzed using an image analyzer Quantity One System (Bio-Rad, CA, USA). The results were expressed as density values normalized to *β*-actin.

### 2.13. Statistical Analysis

Experimental data were expressed as the means ± SEM. Sample number (*n*) is shown in the figure legends. Data were subjected to *t*-test (two groups) or one-way ANOVA (three or more groups) followed by Bonferroni correction for post hoc *t*-test. Probabilities of 0.05 or less were considered statistically significant.

## 3. Results

### 3.1. Effect of Polydatin and DAPT on Heart Function and Myocardial Injury in MI/R-Injured Diabetic Heart

**Figure 1:**
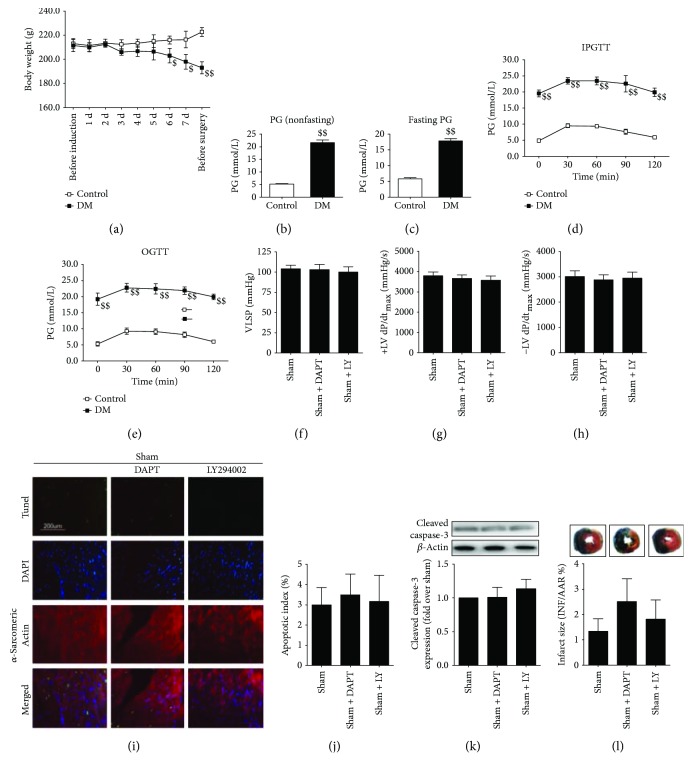
Effect of DAPT and LY294002 on myocardial injury in sham-operated diabetic rats. The diabetic animals received sham operation in the presence or absence of DAPT or LY294002 treatment. (a) Body weight of control rats and diabetic rats. The data was collected before and during the induction of diabetes (7 days). Before the MI/R surgery, the body weight was measured once again. (b) Bar diagram showing nonfasting plasma glucose of control rats and diabetic rats after 7 days of streptozotocin injection. (c) Bar diagram showing fasting plasma glucose of control rats and diabetic rats after 7 days of streptozotocin injection. (d) Intraperitoneal glucose tolerance test after 7 days of streptozotocin injection. (e) Oral glucose tolerance test (OGTT) after 7 days of streptozotocin injection. (f) Bar diagram showing left ventricular systolic pressure. (g) and (h) Bar diagram showing first derivative of left ventricular pressure (+dP/dt_max_ and −dP/dt_max_). (i) Representative photomicrographs of in situ detection of apoptotic myocytes by TUNEL staining (×200, bar = 200 *μ*m). Apoptotic nuclei were stained with TUNEL (row 1, green staining). Total nuclei were stained with DAPI (row 2, blue staining). Cardiomyocytes were stained with anti-*α*-sarcomeric actin (row 3, red staining). (j) Bar diagram showing apoptotic index (percentage of TUNEL-positive nuclei). (k) Bar diagram showing cleaved caspase-3 expression. Top images: representative blots. (l) Bar diagram showing myocardial infarct size. Top images: representative photographs of heart sections. Blue-stained area indicates nonischemic, normal region; red-stained area, ischemia-reperfused but not infarcted region; and negative-stained area, ischemia-reperfused infarcted region. *n* = 6 animals or samples per group. ^$$^*P* < 0.01/^$^*P* < 0.05 versus the control group. DM, diabetes mellitus; PG, plasma glucose; IPGTT, intraperitoneal glucose tolerance test; OGTT, oral glucose tolerance test; MI/R, myocardial ischemia-reperfusion; LY, LY294002; LVSP, left ventricular systolic pressure; AAR, area at risk; INF, infarct area.

**Figure 2:**
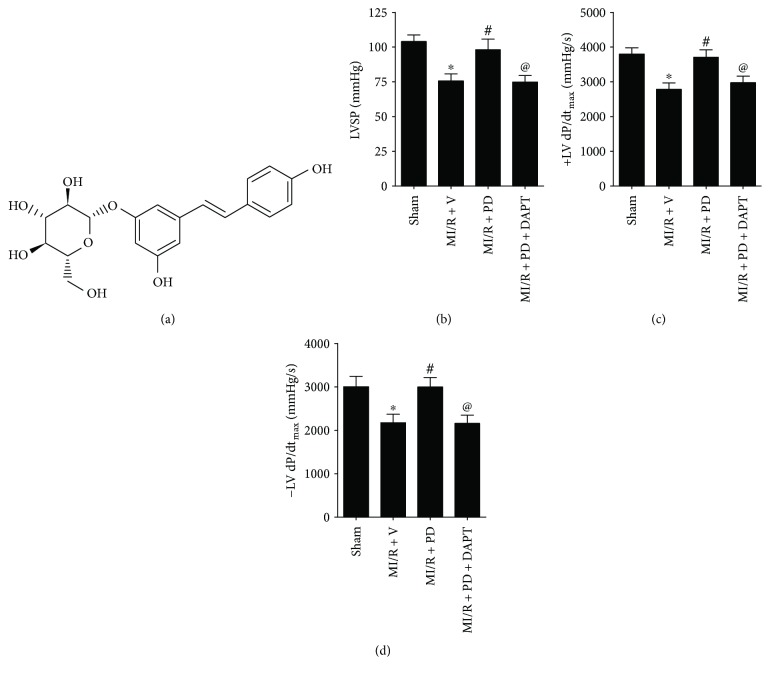
Effect of polydatin and DAPT on cardiac function in MI/R-injured diabetic rats. The diabetic animals received MI/R operation in the presence or absence of polydatin or DAPT treatment. The cardiac function was measured after 3 hours of reperfusion. (a) Chemical structure of polydatin (3,4′,5-trihydroxystilbene-3-*β*-D-glucoside, C_20_H_22_O_8_). (b) Bar diagram showing left ventricular systolic pressure. (c) and (d) Bar diagram showing first derivative of left ventricular pressure (+dP/dt_max_ and −dP/dt_max_). The results are expressed as the mean ± SEM. *n* = 6 animals per group. ^∗^*P* < 0.05 versus the sham group, ^#^*P* < 0.05 versus the MI/R + V group, and ^@^*P* < 0.05 versus the MI/R + PD group. MI/R, myocardial ischemia-reperfusion; V, vehicle; LVSP, left ventricular systolic pressure.

**Figure 3:**
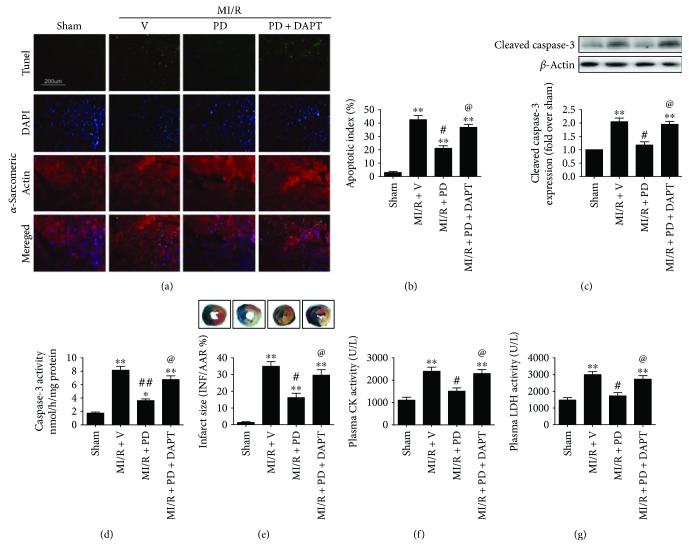
Effect of polydatin and DAPT on cardiac function in MI/R-injured diabetic rats. The diabetic animals received MI/R operation in the presence or absence of polydatin or DAPT treatment. TUNEL staining and Evans blue-TTC double staining were performed after 6 hours of reperfusion. Cleaved caspase-3 expression, caspase-3 activity, and plasma CK and LDH activity measurement were performed after 3 hours of reperfusion. (a) Representative photomicrographs of in situ detection of apoptotic myocytes by TUNEL staining (×200, bar = 200 *μ*m). Apoptotic nuclei were stained with TUNEL (row 1, green staining). Total nuclei were stained with DAPI (row 2, blue staining). Cardiomyocytes were stained with anti-*α*-sarcomeric actin (row 3, red staining). (b) Bar diagram showing apoptotic index (percentage of TUNEL-positive nuclei). (c) Bar diagram showing cleaved caspase-3 expression. Top images: representative blots. (d) Bar diagram showing caspase-3 activity. (e) Bar diagram showing myocardial infarct size. Top images: representative photographs of heart sections. Blue-stained area indicates nonischemic, normal region; red-stained area, ischemia-reperfused but not infarcted region; and negative-stained area, ischemia-reperfused infarcted region. (f) and (g) Bar diagram showing plasma CK and LDH activity. The results are expressed as the mean ± SEM. *n* = 6 samples per group. ^∗∗^*P* < 0.01/^∗^*P* < 0.05 versus the sham group, ^##^*P* < 0.01/^#^*P* < 0.05 versus the MI/R + V group, and ^@^*P* < 0.05 versus the MI/R + PD group. MI/R, myocardial ischemia-reperfusion; V, vehicle; PD, polydatin; AAR, area at risk; INF, infarct area; CK, creatine kinase; LDH, lactate dehydrogenase.

As shown in Figures 1(a)–1(c), diabetic animals exhibited significantly reduced body weight as well as increased nonfasting and fasting plasma glucose (compared with the control group, *P* < 0.05). Then, IPGTT and OGTT were performed. As expected, type 1 diabetic rats showed markedly impaired IPGTT and OGTT (Figures 1(d) and 1(e)). To explore the potential therapeutic effect of polydatin on MI/R injury under diabetic condition, we analyzed the heart function after 3 hours of reperfusion. As shown in Figure 2, compared with the sham group, ischemia-reperfusion injury significantly decreased left ventricular systolic pressure (from 104.0 ± 4.8 to 75.6 ± 5.1, *P* < 0.05) as well as the first derivative of left ventricular pressure (+dP/dt_max_, from 3796 ± 182.1 to 2793 ± 183.5, *P* < 0.05; −dP/dt_max_, from 3006 ± 236.8 to 2174 ± 198.2, *P* < 0.05), indicating that MI/R surgery markedly reduced heart function under diabetic condition. Previously, apoptosis is known to contribute greatly to diabetic MI/R injury [22]. We therefore measured the percentage of TUNEL-positive nuclei and the expression of cleaved caspase-3. As shown in Figures 3(a)–3(d), ischemia-reperfusion injury significantly increased the percentage of TUNEL-positive nuclei and cleaved caspase-3 expression (compared with the sham group, *P* < 0.05). Additionally, the MI/R-injured group also exhibited increased caspase-3 activity, infarct size, and plasma CK and LDH activity (Figures 3(d)–3(g), compared with the sham group, *P* < 0.05). Polydatin is demonstrated to confer myocardial protective effects [17, 18]; however, its effect on MI/R injury in a diabetic state remains unknown. In the present experiment, we found that polydatin administration effectively preserved heart function by increasing left ventricular systolic pressure (Figure 2(b), from 75.6 ± 5.1 to 98.1 ± 7.2, *P* < 0.05) and the first derivative of left ventricular pressure (Figure 2(c), +dP/dt_max_, from 2793 ± 183.5 to 3711 ± 211.5, *P* < 0.05; Figure 2(d), −dP/dt_max_, from 2174 ± 198.2 to 2997 ± 218.4, *P* < 0.05). Additionally, the polydatin-treated group exhibited significantly reduced myocardial apoptosis as evidenced by a decreased percentage of TUNEL-positive nuclei, decreased cleaved caspase-3 expression, and suppressed caspase-3 activity (Figures 3(a)–3(d), compared with the MI/R + V group, *P* < 0.05). Moreover, we found that polydatin also limited myocardial infarction (Figure 3(e), *P* < 0.05) and reduced plasma CK and LDH activity (Figures 3(f) and 3(g), *P* < 0.05).

Recently, Notch1 has attracted increasing attention for its myocardial protective actions [9, 10]. To explore whether Notch1 signaling plays a role in the present circumstance, we employed DAPT (a *γ*-secretase inhibitor), which was widely used to study Notch1 function [11, 23, 26]. Initially, we evaluated the effect of DAPT on a sham-operated heart. We found that DAPT treatment had no significant effect on cardiac function and myocardial apoptosis and infarction (Figures 1(a)–1(g), compared with the sham group, *P* > 0.05). Next, we found that compared with the MI/R + PD group, the DAPT-treated group exhibited significantly decreased left ventricular systolic pressure (Figure 2(b), from 98.1 ± 7.2 to 74.8 ± 4.8, *P* < 0.05) and the first derivative of left ventricular pressure (Figure 2(c), +dP/dt_max_, from 3711 ± 211.5 to 2972 ± 188.9, *P* < 0.05; Figure 2(d), −dP/dt_max_, from 2997 ± 218.4 to 2160 ± 189.6, *P* < 0.05). Moreover, DAPT treatment markedly aggravated myocardial apoptosis as evidenced by an increased percentage of TUNEL-positive nuclei and cleaved caspase-3 expression as well as caspase-3 activity (Figures 3(a)–3(d), compared with the MI/R + PD group, *P* < 0.05). Our data also showed DAPT inhibited the protective effect of polydatin by increasing myocardial infarction and plasma CK and LDH activity (Figures 3(e)–3(g), compared with the MI/R + PD group, *P* < 0.05). These results suggested that short-term polydatin administration effectively reduced diabetic MI/R injury. Additionally, Notch1 signaling probably played a key role in this process.

### 3.2. Effect of Polydatin and DAPT on Myocardial Oxidative Stress in MI/R-Injured Diabetic Heart

**Figure 4:**
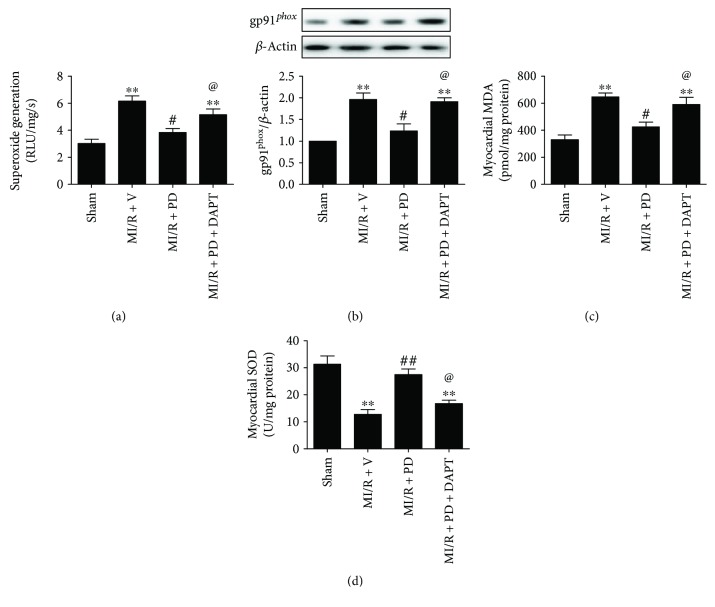
Effect of polydatin and DAPT on myocardial oxidative stress markers in MI/R-injured diabetic rats. The diabetic animals received MI/R operation in the presence or absence of polydatin or DAPT treatment. The measurement of oxidative stress markers was performed after 3 hours of reperfusion. (a) Bar diagram showing cardiac superoxide generation. (b) Bar diagram showing gp91*^phox^* expression. Top images: representative blots. (c) Bar diagram showing myocardial malondialdehyde (MDA) contents. (d) Bar diagram showing myocardial superoxide dismutase (SOD) contents. The results are expressed as the mean ± SEM. *n* = 6 samples per group. ^∗∗^*P* < 0.01/^∗^*P* < 0.05 versus the sham group, ^##^*P* < 0.01/^#^*P* < 0.05 versus the MI/R + V group, and ^@@^*P* < 0.01/^@^*P* < 0.05 versus the MI/R + PD group. MI/R, myocardial ischemia-reperfusion; V, vehicle; PD, polydatin.

We and others have shown that oxidative stress played a key role in MI/R injury [21–23]. In addition, Miao et al. demonstrated that polydatin exerted an antioxidative effect during MI/R injury [27]. However, its actions in the diabetic state and the underlying mechanisms remain unknown. In cardiomyocyte, gp91*^phox^* is a major component of NADPH oxidase and is also deemed as an important oxidative stress marker. As shown in Figures 4(a) and 4(b), ischemia-reperfusion injury greatly enhanced myocardial oxidative stress by increasing superoxide generation and gp91*^phox^* expression (compared with the sham group, *P* < 0.05). Moreover, cardiac MDA level was significantly increased, while SOD activity was markedly decreased in the MI/R + V group (Figures 4(c) and 4(d), compared with the sham group, *P* < 0.05). Intriguingly, polydatin treatment inhibited myocardial oxidative stress by reducing superoxide accumulation, gp91*^phox^* expression, and MDA level while increasing myocardial SOD activity (Figures 4(a)–4(d), compared with the MI/R + V group, *P* < 0.05). Next, we evaluated whether Notch1 participated in this process. We found that the suppressive effects of polydatin on myocardial oxidative damage were inhibited by DAPT treatment as evidenced by increased superoxide accumulation, gp91*^phox^* expression, and MDA level as well as decreased myocardial SOD activity (Figures 4(a)–4(d), compared with the MI/R + PD group, *P* < 0.05). These data indicated that Notch1 signaling contributed greatly to the antioxidative effect of polydatin against MI/R injury in the diabetic state.

### 3.3. Effect of Polydatin and DAPT on Myocardial Nitrative Stress in MI/R-Injured Diabetic Heart

**Figure 5:**
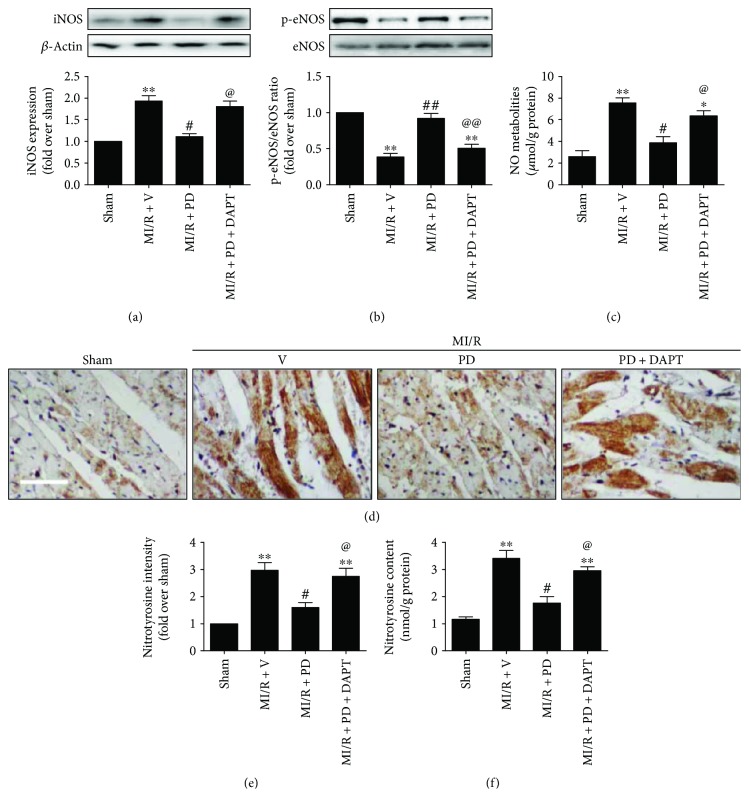
Effect of polydatin and DAPT on myocardial nitrative stress in MI/R-injured diabetic rats. The diabetic animals received MI/R operation in the presence or absence of polydatin or DAPT treatment. The measurement was performed after 3 hours of reperfusion. (a) Bar diagram showing iNOS expression. Top images: representative blots. (b) Bar diagram showing eNOS phosphorylation level. Top images: representative blots. (c) Bar diagram showing NO metabolites level. (d) Representative myocardial immunohistochemistry images of nitrotyrosine (×200, bar = 200 *μ*m). (e) Bar diagram showing nitrotyrosine intensity. (f) Bar diagram showing myocardial nitrotyrosine content. The results are expressed as the mean ± SEM. *n* = 6 samples per group. ^∗∗^*P* < 0.01/^∗^*P* < 0.05 versus the sham group, ^##^*P* < 0.01/^#^*P* < 0.05 versus the MI/R + V group, and ^@@^*P* < 0.01/^@^*P* < 0.05 versus the MI/R + PD group. MI/R, myocardial ischemia-reperfusion; V, vehicle; PD, polydatin.

Next, we evaluated the potential effect of polydatin on cardiac nitrative stress induced by MI/R injury in the diabetic state. As depicted in Figure 5, the NO metabolites were significantly increased in the MI/R + V group (7.58 ± 0.45 versus 2.61 ± 0.54, compared with the MI/R + PD group, *P* < 0.05), while the phosphorylation level of eNOS (at serine 1177) was markedly suppressed by MI/R injury (compared with the MI/R + PD group, *P* < 0.05). It has been established that NO, produced from L-arginine by a family of NO synthase (NOS), is an important mediator in myocardial infarction [28]. The activation of eNOS can enhance myocardial NO production, which seems to be inconsistent with our data. However, this paradoxical result indicated that other forms of NO synthase probably played a central role in this process. Therefore, we measured the protein level of iNOS in the myocardium. Our Western blotting results showed that MI/R injury markedly increased iNOS expression (Figure 5(a), compared with the sham group, *P* < 0.05). Next, we measured myocardial nitrotyrosine level (an index for nitrative stress). As shown in Figures 5(d)–5(f), both immunohistochemical results and ELISA results showed that the nitrotyrosine level was significantly increased in the MI/R-injured group (compared with the sham group, *P* < 0.05). Intriguingly, polydatin treatment exhibited an antioxidative effect as evidenced by reduced tissue nitrotyrosine level (Figures 5(d)–5(f), compared with the MI/R + V group, *P* < 0.05). We therefore examined whether polydatin affected the NO metabolites and NO synthase expression. Our data showed that polydatin significantly reduced the production of NO metabolites while it increased p-eNOS/eNOS ratio (Figures 5(b) and 5(c), compared with the MI/R + V group, *P* < 0.05), suggesting that iNOS might play a key role in the present circumstance. As expected, we found that iNOS expression was markedly reduced by polydatin treatment (Figure 5(a), compared with the MI/R + V group, *P* < 0.05). These results suggested that suppression of iNOS expression contributed greatly to the antinitrative effect of polydatin during MI/R injury in the diabetic state.

As previous publications have indicated that Notch1 activation inhibited myocardial nitrative stress [13], we proceeded to investigate the possible involvement of Notch1 in polydatin's myocardial protective actions. As shown in Figures 5(a) and 5(b), the DAPT-treated group showed significantly increased iNOS expression and decreased eNOS phosphorylation (compared with the MI/R + PD group, *P* < 0.05). Additionally, the suppressive effect of polydatin on NO metabolites and nitrotyrosine was also inhibited by DAPT treatment (Figures 5(c)–5(f), compared with the MI/R + PD group, *P* < 0.05). These data suggested that Notch1 signaling played a key role in polydatin's antinitrative effect.

### 3.4. Effect of Polydatin and DAPT on Notch1/Hes1 Signaling in MI/R-Injured Diabetic Heart

**Figure 6:**
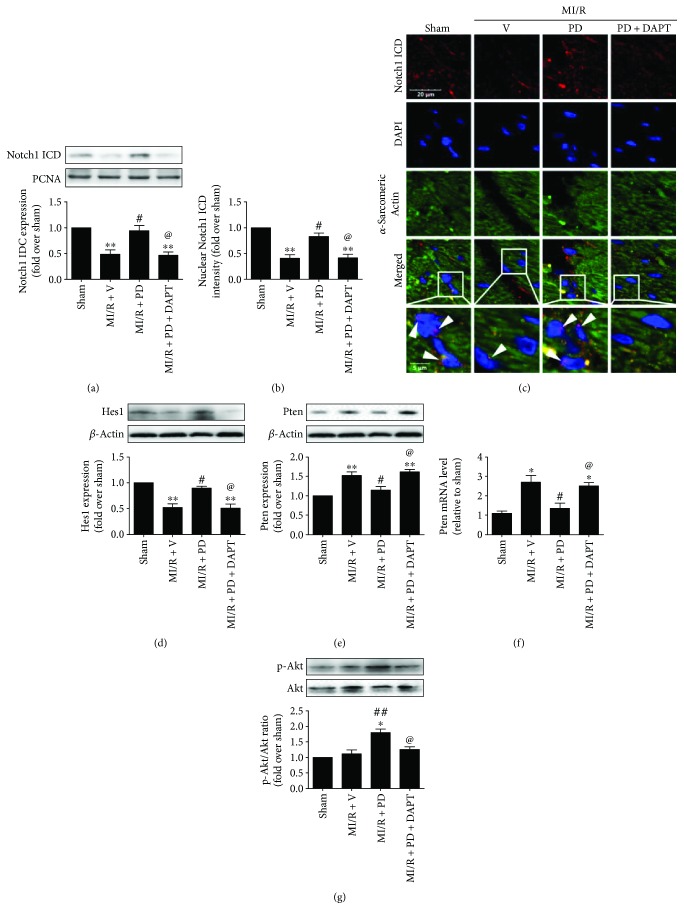
Effect of polydatin and DAPT on myocardial Notch1/Hes1 signaling in MI/R-injured diabetic rats. The diabetic animals received MI/R operation in the presence or absence of polydatin or DAPT treatment. The assessment was performed after 3 hours of reperfusion. (a) Bar diagram showing Notch1 ICD nuclear expression. Top images: representative blots. (b) Bar diagram showing nuclear Notch1 ICD intensity. (c) Representative photomicrographs of Notch1 ICD immunofluorescent staining (bar = 5 *μ*m). Red fluorescence shows Notch1 ICD (row 1). Blue fluorescence shows cardiomyocyte nuclei (row 2). Green fluorescence shows cardiomyocytes (*α*-sarcomeric-actin, row 3). Arrows show Notch1 ICD nuclear localization; original magnification ×400. (d) Bar diagram showing Hes1 expression. Top images: representative blots. (e) Bar diagram showing Pten expression. Top images: representative blots. (f) Bar diagram showing real-time PCR analysis of Pten mRNA level in the myocardium. (g) Bar diagram showing Akt phosphorylation. Top images: representative blots. The results are expressed as the mean ± SEM. *n* = 6 samples per group. ^∗∗^*P* < 0.01/^∗^*P* < 0.05 versus the sham group, ^##^*P* < 0.01/^#^*P* < 0.05 versus the MI/R + V group, and ^@^*P* < 0.05 versus the MI/R + PD group. MI/R, myocardial ischemia-reperfusion; V, vehicle; PD, polydatin.

To further confirm the underlying mechanism, we studied myocardial Notch1 and its downstream signaling molecules. The nuclear distribution of Notch1 ICD was investigated by Western blotting as well as immunofluorescent staining and used as the marker of Notch1 activation. As depicted in Figure 6(a), we found a markedly suppressed nuclear Notch1 ICD expression in the MI/R-injured myocardium (compared with the sham group, *P* < 0.05). The result of immunofluorescence also showed that Notch1 ICD distribution was decreased in the MI/R + V group (Figures 6(b) and 6(c), compared with the sham group, *P* < 0.05). Additionally, ischemia-reperfusion injury suppressed the expression of Hes1 while it enhanced the Pten expression (Figures 6(d)–6(f), compared with the sham group, *P* < 0.05), indicating that the myocardial Notch1/Hes1 signaling pathway was reduced in a reperfused heart. However, these effects were inhibited by polydatin treatment as evidenced by increased Notch1 ICD nuclear distribution and Hes1 expression and decreased Pten level (Figures 6(a)–6(f), compared with the MI/R + V group, *P* < 0.05). Moreover, polydatin also enhanced Akt signaling by increasing its phosphorylation level (Figure 6(g), compared with the MI/R + V group, *P* < 0.05). Finally, we found that DAPT effectively inhibited Notch1 signaling by reducing Notch1 ICD nuclear expression and Hes1 level (Figures 6(a)–6(d), compared with the MI/R + PD group, *P* < 0.05). The modulatory effect of polydatin on Pten and Akt was also suppressed by DAPT administration as evidenced by markedly increased Pten expression and reduced Akt phosphorylation (Figures 6(e)–6(g), compared with the MI/R + PD group, *P* < 0.05). These data indicate that polydatin exerts cardioprotection against diabetic MI/R injury by activating myocardial Notch1/Hes1 signaling. Moreover, Pten-Akt signaling probably served as the downstream signaling pathway.

### 3.5. Effect of LY294002 on Heart Function, Myocardial Apoptosis, and Infarction in MI/R-Injured Diabetic Heart

**Figure 7:**
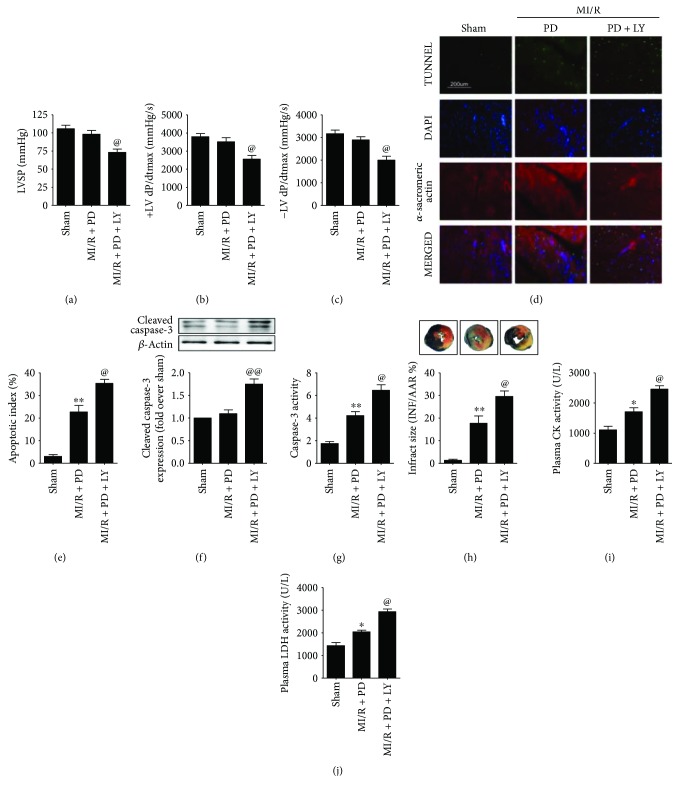
Effect of LY294002 on cardiac function and myocardial damage in MI/R-injured diabetic rats. TUNEL staining and Evans blue-TTC double staining were performed after 6 hours of reperfusion. The rest of the measurements was carried out after 3 hours of reperfusion. (a) Bar diagram showing left ventricular systolic pressure. (b) and (c) Bar diagram showing first derivative of left ventricular pressure (+dP/dt_max_ and −dP/dt_max_). (d) Representative photomicrographs of in situ detection of apoptotic myocytes by TUNEL staining (×200, bar = 200 *μ*m). Apoptotic nuclei were stained with TUNEL (row 1, green staining). Total nuclei were stained with DAPI (row 2, blue staining). Cardiomyocytes were stained with anti-*α*-sarcomeric actin (row 3, red staining). (e) Bar diagram showing apoptotic index (percentage of TUNEL-positive nuclei). (f) Bar diagram showing cleaved caspase-3 expression. Top images: representative blots. (g) Bar diagram showing caspase-3 activity. (h) Bar diagram showing myocardial infarct size. Top images: representative photographs of heart sections. Blue-stained area indicates nonischemic, normal region; red-stained area, ischemia-reperfused but not infarcted region; and negative-stained area, ischemia-reperfused infarcted region. (i) and (j) Bar diagram showing plasma CK and LDH activity. The results are expressed as the mean ± SEM. *n* = 6 animals or samples per group. ^∗∗^*P* < 0.01/^∗^*P* < 0.05 versus the sham group and ^@@^*P* < 0.01/^@^*P* < 0.05 versus the MI/R + PD group. MI/R, myocardial ischemia-reperfusion; PD, polydatin; LY, 294002; LVSP, left ventricular systolic pressure; AAR, area at risk; INF, infarct area; CK, creatine kinase; LDH, lactate dehydrogenase.

Intracellular phosphatidylinositol-3-kinase (PI3K)/Akt signaling, which regulates a vast array of cellular processes involved in the cell cycle, has been well established as the pivotal survival signaling in cardiomyocytes [29]. To explore the downstream signaling of Notch1 in polydatin's ameliorative effect against diabetic MI/R injury, we studied Akt signaling. Compared with the sham group, LY294002 alone had no significant effect on the heart function (Figures 1(a)–1(c), *P* > 0.05), apoptosis (Figures 1(d)–1(f), *P* > 0.05), and myocardial infarction (Figure 1(g), *P* > 0.05) in a sham-operated diabetic heart. However, we found a significant decrease in left ventricular systolic pressure (Figure 7(a), from 98.1 ± 7.2 to 73.2 ± 4.5, *P* < 0.05) and the first derivative of left ventricular pressure (Figures 7(b) and 7(c), +dP/dt_max_, from 3711 ± 211.5 to 2659 ± 230.0, *P* < 0.05; −dP/dt_max_, from 2997 ± 218.4 to 1998 ± 179.0, *P* < 0.05) in the LY294002-treated group (compared with the MI/R + PD group). LY294002 treatment also blunted the antiapoptotic effect of polydatin as evidenced by an increased percentage of TUNEL-positive nuclei (Figures 7(d) and 7(e), compared with the MI/R + PD group, *P* < 0.05) and upregulated cleaved caspase-3 expression as well as caspase-3 activity (Figures 7(f) and 7(g), compared with the MI/R + PD group, *P* < 0.05). Additionally, myocardial infarction was also aggravated by LY294002 administration as evidenced by increased infarct size (Figure 7(h), compared with the MI/R + PD group, *P* < 0.05) and plasma CK and LDH activities (Figures 7(i) and 7(j), compared with the MI/R + PD group, *P* < 0.05). These data suggested that Akt activation contributed greatly to the protective effect of polydatin against diabetic MI/R injury.

### 3.6. Effect of LY294002 on Myocardial Oxidative Stress and Nitrative Stress in MI/R-Injured Diabetic Heart

**Figure 8:**
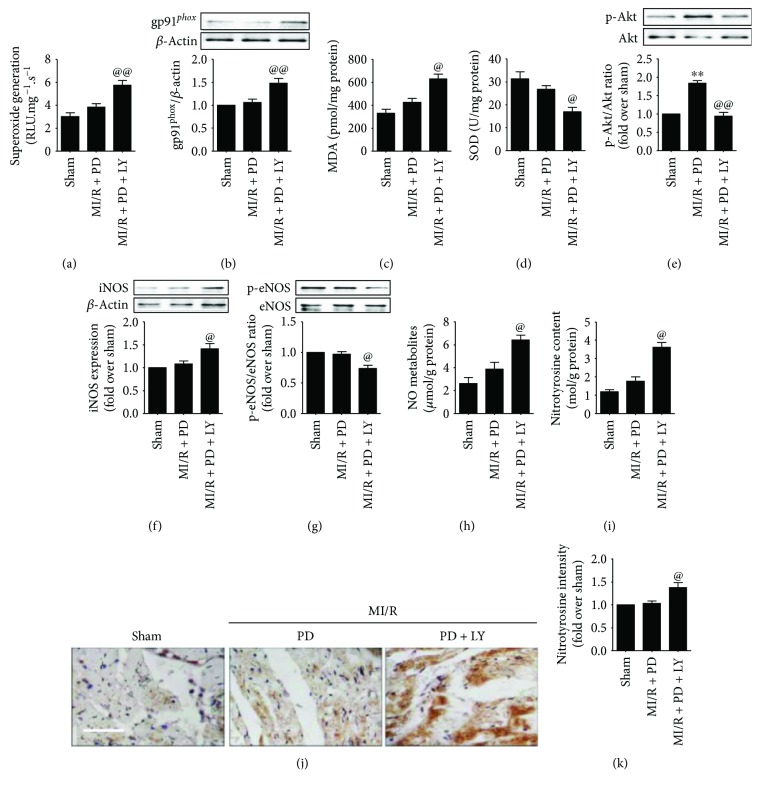
Effect of LY294002 on myocardial oxidative/nitrative stress in MI/R-injured diabetic rats. The measurement was performed after 3 hours of reperfusion. (a) Bar diagram showing cardiac superoxide generation. (b) Bar diagram showing gp91*^phox^* expression. Top images: representative blots. (c) Bar diagram showing myocardial malondialdehyde (MDA) contents. (d) Bar diagram showing myocardial superoxide dismutase (SOD) contents. (e) Bar diagram showing Akt phosphorylation. Top images: representative blots. (f) Bar diagram showing iNOS expression. Top images: representative blots. (g) Bar diagram showing eNOS phosphorylation level. Top images: representative blots. (h) Bar diagram showing NO metabolites level. (i) Bar diagram showing nitrotyrosine intensity. (j) Representative myocardial immunohistochemistry images of nitrotyrosine (×200, bar = 200 *μ*m). (k) Bar diagram showing myocardial nitrotyrosine content. The results are expressed as the mean ± SEM. *n* = 6 samples per group. ^∗∗^*P* < 0.01/^∗^*P* < 0.05 versus the sham group and ^@@^*P* < 0.01/^@^*P* < 0.05 versus the MI/R + PD group. MI/R, myocardial ischemia-reperfusion; PD, polydatin; LY, 294002.

Previous publications have demonstrated that Akt signaling regulated myocardial oxidative/nitrative stress during ischemia-reperfusion injury [29, 30]. However, its role in the myocardial protective effect of polydatin remains poorly defined. In the present study, we found that the antioxidative effect of polydatin was blunted by Akt inhibition. As shown in Figures 8(a)–8(d), the LY-treated group exhibited markedly increased superoxide generation, gp91*^phox^* expression as well as myocardial MDA level, and decreased SOD activity (compared with the MI/R + PD group, *P* < 0.05). Additionally, LY294002 not only inhibited the stimulatory effect of polydatin on myocardial Akt signaling (Figure 8(e), compared with the MI/R + PD group, *P* < 0.05) but also upregulated iNOS expression and NO metabolites while it downregulated eNOS phosphorylation (Figures 8(f)–8(h), compared with the MI/R + PD group, *P* < 0.05). Moreover, tissue nitrotyrosine level was significantly increased by LY294002 treatment (Figures 8(i)–8(k), compared with the MI/R + PD group, *P* < 0.05). These data suggested that Akt signaling played a central role in the antioxidative and antinitrative effect of polydatin against diabetic MI/R injury.

## 4. Discussion

In this study, we found that polydatin exerts a profound myocardial protective effect against ischemia-reperfusion injury by reducing oxidative stress and nitrative stress under diabetic condition. Additionally, polydatin treatment significantly enhanced Notch1/Hes1 signaling and further activated the downstream Pten/Akt pathway. Inhibiting either Notch1 signaling or Akt pathway almost blocked its therapeutic effect. We demonstrated for the first time that polydatin suppressed myocardial oxidative/nitrative stress damage by targeting on Notch1/Hes1 signaling. Furthermore, we proposed that Notch1/Hes1 and its downstream Pten/Akt pathway might serve as novel therapeutic targets for treating ischemic heart disease in diabetic setting.

Diabetes has been considered as a major risk factor for cardiovascular disease. Numerous clinical and animal studies have shown that diabetic subjects have significantly more severe and fatal myocardial infarctions than nondiabetic subjects [5, 21, 31–33]. Considerable evidence indicates that hyperglycemia and hyperlipidemia not only cause vascular injury that leads to ischemic heart disease but also have a direct adverse impact on ischemic cardiomyocytes, which results in larger infarct size after reperfusion therapy [23, 34, 35]. Safe and effective approaches against myocardial ischemia-reperfusion (MI/R) injury and cellular apoptosis may provide us a better outcome in the management of diabetes-related heart disease [2, 36]. Previously, we and others have confirmed that enhanced oxidative stress and nitrative stress during acute reperfusion period are the crucial contributors to MI/R injury [13, 22, 37]. Oxidative/nitrative stress is defined as an excessive accumulation or insufficient removal of highly reactive molecules such as ROS and/or reactive nitrogen species (RNS), including superoxide anion, hydroxyl radical, hydrogen peroxide, and peroxynitrite (ONOO^−^) [13, 38]. Indeed, during the ischemia-reperfusion period, cardiac nicotinamide adenine dinucleotide phosphate (NADPH) oxidase serves as the main source of ROS, while peroxynitrite, generated from the reaction of superoxide anion with NO, is proven to be the highly cytotoxic RNS [13]. In the myocardium, NO is synthesized from its precursor L-arginine mainly by a family of NO synthase (NOS), including inducible NOS (iNOS) and endothelial NOS (eNOS) [28, 29]. Depending on the NOS involved, NO could exhibit diverse effects on the heart. Endothelial NOS is localized in caveolae where it controls heart rate, contraction, and diastolic relaxation. However, iNOS is not present in healthy hearts but is highly activated under pathological conditions, including ischemia, hyperglycemia, and inflammation [28]. It has been demonstrated that pathological activation of iNOS generates 100–1000-fold more NO than does eNOS. Low levels of NO exert a beneficial effect in the heart, while higher levels are potentially toxic [39]. During MI/R injury, the excessive NO production for a prolonged period of time exerts detrimental effects. Zaman et al. showed that iNOS inhibition contributed greatly to the beneficial effect of ischemic postconditioning during MI/R injury, and aminoguanidine a selective iNOS inhibitor mimicked this effect [40]. Zhu et al. also found that inhibition of MI/R-induced iNOS reduced endothelial cell migration and apoptosis, thus ameliorating myocardial injury [41]. On the other hand, one recent study demonstrated that recombinant human relaxin-2 (serelaxin) inhibited myocardial infarction following ischemia-reperfusion injury by eNOS activation, while eNOS deletion abolished serelaxin's beneficial effects [42]. In the present study, our data showed that, under diabetic condition, reperfusion injury enhanced oxidative stress markers. Additionally, MI/R injury differentially modulated eNOS and iNOS activities as evidenced by decreased eNOS phosphorylation and increased iNOS expression, eventually aggravating cardiac damage. Based on the above literature and the previous observations in a diabetic heart [43–45], those drugs that increase eNOS activity while reducing iNOS activity to maintain a moderate level of NO serve as critical cardioprotective agents against MI/R injury in diabetic setting.

In this study, we found that polydatin administration effectively ameliorated MI/R injury by reducing myocardial infarction and apoptosis and preserving left ventricular function under the diabetic state. To the best of our knowledge, this is the first study demonstrating the beneficial effect of polydatin against diabetic MI/R injury. Furthermore, our data showed that polydatin not only reduced oxidative stress but also inhibited nitrative stress by increasing p-eNOS/eNOS ratio and decreasing iNOS, myocardial NO metabolites, and nitrotyrosine levels. Previously, several studies have indicated the antioxidant property of polydatin in the cardiovascular system [46, 47]. Moreover, NO synthase was reported to contribute to polydatin's cardiovascular pharmacological actions. Ma et al. showed that polydatin inhibited hydrogen peroxide-induced proliferation of vascular smooth muscle cells (VSMCs) by activating the eNOS/SIRT1 pathway, thus exerting a potential therapeutic effect against vascular plaque formation and atherosclerosis [47]. Another study demonstrated that polydatin exerted a protective effect against myocardial hypertrophy in type 1 diabetic mice via inhibiting the expressions of NF-*κ*B p65, COX-2 and, importantly, iNOS [20]. These data all supported our observations in the present study. Hence, we propose that the antioxidative/nitrative effect of polydatin contributed greatly to its cardiovascular pharmacological actions under diabetic condition.

To validate our hypothesis, myocardial Notch1 signaling was investigated. It has been found that Notch signalings (e.g., Notch1/Hes1) decline steadily in a postnatal heart as the myocardium matures to the adult hormonal and contractile state [48, 49], which may underlie its loss of control in tissue homeostasis during adult stage. However, reexpression of Notch signals has also been found to constitute an adaptive response following pathological insult to increase survival rate or regenerate tissues [49, 50]. For example, pharmacological blockade of Notch1 leads to caspase-3-dependent apoptosis in adult islets; in contrast, overactivation of Notch1 protects against apoptosis [51]. Previously, we and others found that the Notch1/Hes1 pathway is specifically involved in cellular oxidative/nitrative stress regulation [11–13, 23]. The study by Pei and coworkers firstly showed that activation of endogenous Notch1 is critical to promote cardiomyocyte survival and sustain cardiac function after ischemia-reperfusion injury [13]. Another study by Zhang et al. also demonstrated that 2,3,5,4′-tetrahydroxystilbene-2-O-*β*-D-glucoside, an active component from *Polygonum multiflorum* Thunb., ameliorates MI/R injury by activating Notch1 signaling [24]. However, to our knowledge, there are a few studies exploring the role of Notch1 signaling on MI/R injury in diabetic setting. Intriguingly, a recent study by our group found that Notch1/Hes1 activation preserved thioredoxin activity and reduced MI/R injury in an acute hyperglycemic animal model, indicating that Notch1/Hes1 signaling might play a role in ischemic heart disease under diabetic condition [23]. In the present study, we found that ischemia-reperfusion operation significantly inhibited Notch1/Hes1 signaling by decreasing Notch1 ICD nuclear distribution and Hes1 expression. These observations were consistent with the previous in vitro data [26]. Moreover, polydatin administration reactivated Notch1/Hes1 signaling, while inhibition of Notch1 signaling not only blocked polydatin's antiapoptotic effect but also abolished the antioxidative/nitrative effects. These results suggested that the Notch1/Hes1 signaling pathway played a key role in mediating the protective actions of polydatin against diabetic MI/R injury.

We next investigated phosphatase and tensin homolog deleted on chromosome 10 (Pten)/Akt signaling to further explore the underlying mechanism. Previous studies have suggested that the Pten/Akt pathway serves as a direct downstream effector of Notch1/Hes1 signaling [12, 13, 52]. Although the mechanism by which Akt activity is influenced by Notch1 has not been fully elucidated, existing data indicate that Notch1 stimulates Akt activation through suppressing Pten, a renowned tumor suppressor. It has been found that Hes1 could bind to Pten promoter and induce a significant reduction in the activity of the Pten promoter [53, 54]. Moreover, phosphatidylinositol 3,4,5-trisphosphate (PIP3) is the major second messenger of the PI3K pathway that mediates the activation of Akt [55]. Pten negatively regulates the Akt activity by converting PIP3 to phosphatidylinositol 4,5-bisphosphate (PIP2), thus functioning as the suppressor of the PI3K/Akt pathway [55]. Pei and coworkers demonstrated that Notch1 inhibition increased Pten expression and decreased Akt activity in a reperfused myocardium [13]. Our results were consistent with the above observations. We found that Pten/Akt signaling also serves as the downstream target of polydatin in treating MI/R injury under diabetic condition, since polydatin administration decreased Pten expression and increased Akt phosphorylation. Moreover, we demonstrated that this effect was abolished by either DAPT or LY294002 treatment, suggesting that Pten/Akt signaling not only acts as the effector of the Notch1/Hes1 pathway but also plays a critical role in polydatin's therapeutic effect. Intriguingly, as the crucial intracellular survival signaling, Akt signaling was also implicated to regulate myocardial oxidative and nitrative stress during MI/R injury. Previous studies by our group showed that inhibition of the PI3K/Akt pathway by LY294002 markedly aggravated myocardial oxidative damage and exacerbated ischemia-reperfusion injury [23, 56]. Additionally, Akt activation was found to reverse the eNOS/iNOS expression imbalance and reduce nitrative stress injury in the ischemic heart [13, 57]. As expected, similar results were observed in diabetic setting [58, 59]. Therefore, the potential role of Akt signaling in regulating oxidative/nitrative stress was investigated in this study. Our results further confirmed that Akt contributed greatly to the antioxidant and antinitrative effect of polydatin since LY294002 almost blocked these beneficial effects.

This study proposed a potential therapeutic strategy for the treatment of MI/R injury in the diabetic state. However, there are still more to explore behind these experiment data. Firstly, although plenty of clinical studies have been performed to evaluate the beneficial effect of polydatin on human [60, 61], as far as we know, a few of them are focused on diabetes. Since several basic studies, including the present study, have investigated the therapeutic effect of polydatin on diabetes complication [62, 63]; it is of great necessity to perform clinical investigation. Further research is definitely needed before a conclusion can be made. Secondly, our study is limited to STZ-induced type 1 diabetic animals. Although this is a widely-used animal model in diabetes research [64], it is important to evaluate the therapeutic effect of polydatin under type 2 diabetic condition since type 2 DM is the most common form of diabetes, accounting for >90% of all cases. Thirdly, one study by Kerr et al. demonstrated that Akt/mTOR axis controls endothelial Jagged1 expression and, thereby, Notch signalling in vascular smooth muscle cells [65]. While another study showed that Jagged1 combined with androgen receptor could increase Akt phosphorylation, in turn, phosphorylated Akt further regulated cyclin B1 in prostate cancer cells [66]. Although plenty of studies have demonstrated that Akt signaling served as a crucial downstream effector of the Notch1/Hes1 pathway, a cross talk might exist between Akt and Notch1 signaling. During MI/R injury, the possible interplay between Akt and Notch1 pathways has not been investigated. It is of great interest to futher explore their detailed mechanisms under diabetic condition.

## 5. Conclusions

**Figure 9:**
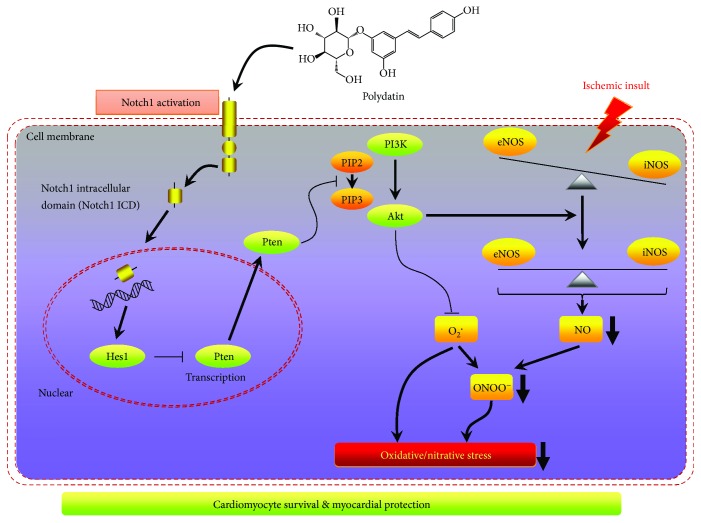
Schematic diagram depicting polydatin's protective mechanisms against diabetic MI/R injury.

Taken together, we showed that polydatin protected against diabetic MI/R injury by reducing oxidative/nitrative stress damage. Moreover, Notch1/Hes1-mediated activation of Pten/Akt signaling played a crucial role in this process (Figure 9). These results reveal that polydatin may be a promising candidate for the treatment of myocardial ischemia-reperfusion injury in cardiac surgery and ischemic heart diseases under diabetic condition. The present study presents critical experimental evidence for designing clinical trials on polydatin supplementation as an early intervention to attenuate MI/R injury in patients with diabetes mellitus.
